# First Trimester DEX Treatment Is Not Associated with Altered Brain Activity During Working Memory Performance in Adults

**DOI:** 10.1210/clinem/dgaa611

**Published:** 2020-09-01

**Authors:** Annelies van’t Westeinde, Marius Zimmermann, Valeria Messina, Leif Karlsson, Nelly Padilla, Svetlana Lajic

**Affiliations:** 1 Department of Women’s and Children’s Health, Karolinska Institutet, Pediatric Endocrinology Unit (QB83), Karolinska University Hospital, Stockholm, Sweden; 2 Section for Cognitive Systems, DTU Compute, Technical University of Denmark Lyngby, Denmark; 3 Department of Women’s and Children’s Health, Karolinska Institutet, Department of Neonatology Norrbacka, Karolinska vägen, Sweden

**Keywords:** dexamethasone, working memory, brain function, cortisol, prenatal glucocorticoid treatment

## Abstract

**Context:**

Prenatal dexamethasone (DEX) treatment is sometimes used in pregnancies at risk for congenital adrenal hyperplasia (CAH) to prevent virilization in female fetuses with CAH. In boys and in fetuses not having CAH, there is no benefit of early DEX treatment and the risks of this therapy must be thoroughly investigated. High doses of prenatal glucocorticoid might alter the developmental trajectory of the brain into adulthood, even for CAH unaffected subjects treated with DEX for a short term during the first trimester.

**Objective:**

The present study investigated brain activation during working memory performance in DEX-treated subjects compared with controls.

**Design, Setting, and Participants:**

We tested 18 participants who were exposed to DEX during the first trimester of fetal life but did not have CAH (8 females; mean age 20.78 [standard deviation (SD), 2.67] years) and 40 control participants (24 females; mean age 20.53 [SD, 2.64]) from a single research institute. Participants underwent functional magnetic resonance imaging on a 3T scanner during a verbal and visuospatial working memory task.

**Results:**

We did not observe any differences in brain activity during working memory performance. However, DEX-treated subjects responded faster during the experimental condition of the verbal WM task.

**Conclusions:**

First trimester DEX treatment did not seem to result in altered working memory–related brain activity at adult age. Our findings contribute to the risk–benefit assessment of prenatal DEX treatment in the context of CAH.

Congenital adrenal hyperplasia (CAH) is a genetic disorder caused by mutations in the *CYP21A2* gene, resulting in a defective 21-hydroxylase enzyme necessary to produce cortisol and aldosterone (1). Life-long replacement therapy with glucocorticoids and mineralocorticoids must be initiated during the second week of life to prevent salt-losing crises (2). Prenatally, the lack of cortisol results in overproduction of adrenal androgens due to the negative feedback loop on the HPA (hypothalamic–pituitary–adrenal) axis. If the fetus with CAH is female, this will lead to in utero virilization, and potentially more male-stereotypical play behavior in childhood (3). As the genitalia begin to differentiate already in gestational week (GW) 7, treatment with the synthetic glucocorticoid dexamethasone (DEX) to prevent virilization needs to be started before that time point to be effective (4). However, as the mutation is inherited in a recessive way, only 1 out of 4 pregnancies from parents who are carriers for CAH will be a fetus with CAH. Further, as only girls with CAH benefit from the treatment, this leads to 1 out of 8 pregnancies requiring DEX treatment until term. In most countries, prenatal genotyping for CAH is not possible until GWs 10 to 12; hence, this practice results in unneeded treatment of 7 out of 8 pregnancies, namely those who do not have CAH, or are boys. Recently the clinical guidelines have been altered and no longer recommend the practice outside of research settings (5). The glucocorticoid dose to which the DEX-treated fetus is exposed is very high, 60 times higher than the physiological level of cortisol during that fetal period (6, 7), and the long-term consequences of the treatment have not been fully elucidated. Previous research on prenatal betamethasone treatment during the third trimester and studies on naturally varying prenatal maternal cortisol levels during both second and third trimester have demonstrated the impact of altered prenatal cortisol on the development of both structure and function of the brain, well into childhood and adolescence (8-12). However, first-trimester cortisol exposure studies are so far lacking. Results from our follow-up study in Sweden on the psychological and somatic effects of pre- and postnatal glucocorticoid treatment show that, although impairments in cognition are found in DEX-treated children compared with healthy controls, these differences seem to attenuate at adult age in children not having CAH (13-15). However, in DEX-treated healthy/non-CAH adults we did observe alterations in the brain of both gray matter and white matter (WM), which included bilateral enlargement of the amygdala and enlarged left superior frontal gyrus (SFg) (16). Notably, the observed changes in structure did not correlate with cognitive performance or symptoms of anxiety and depression. Thus, despite altered structure of the brain at adult age, healthy DEX-treated subjects performed similar to nontreated controls in terms of cognition and behavior. We therefore sought to investigate whether cognitive performance in DEX-treated subjects is associated with alterations in functional activation of the brain that might account for a potential compensatory mechanism resulting in normalized cognitive performance. The present study therefore investigated functional activity of the brain during a verbal and visuospatial working memory task in DEX-treated healthy/non-CAH subjects, and population controls. The results will contribute to our understanding of the impact of first-trimester DEX treatment on the development of the brain until adult age. Understanding of such alterations in brain function contributes to the assessment of the risks and benefits of DEX treatment in pregnancies.

## Patients and Methods

### Subjects

Participants were part of a longitudinal study on CAH and on prenatal DEX treatment, which have been previously described in detail (14, 17). In the prenatal DEX treatment program for pregnancies at risk of CAH, a chorionic villous biopsy is performed at gestational weeks 10 to 12 to genotype for 21-hydroxylase deficiency. DEX treatment is then stopped if the fetus either does not have CAH, or is a CAH boy. All babies in Sweden are further screened for CAH by measuring blood level 17OHP at 2 days of age. In the present report, we included subjects that had been treated prenatally with DEX during the first trimester of fetal life because they were at risk of having CAH due to an earlier CAH pregnancy of the mother (range start DEX: GW 5-9, range stop DEX: GW 10-22, mean duration DEX: 6 weeks [range 1.5-14 weeks]) and who did not have CAH, and untreated controls (C) from the general Swedish population. Subjects were excluded if they had a history of or were currently experiencing neuropsychological problems or treatments affecting the nervous system (5 controls). Further, 3 control subjects and 1 DEX-treated subject were excluded from the analyses because of excessive head movements during the scanning sessions (ie, head displacement >3 mm). One participant was excluded due to ventricular dilation, and 1 participant was excluded due to signal loss in the frontal cortex related to metallic braces. The analyses are thus performed on 18 DEX-treated subjects (8 females, 10 males), mean age 20.78 (2.67) years, and 40 controls (24 females, 16 males), mean age 20.53 (2.64) years. In the control group, 2 subjects had Arnold–Chiari malformation type 1, 4 had minor WM changes, and 2 had a Rathke cyst. In the DEX-treated group, 1 subject had a corpus pineal cyst. Demographic information is presented in Table 1. There were no significant differences between DEX-treated subjects and controls in age, education, parental education, wellbeing, alcohol and drug use, and smoking. All participants, and parents of minors, gave their written informed consent to take part in the study. The study was approved by the Regional Ethical Committee of Karolinska Institutet (no.99-153 and 1658-32).

**Table 1.** Demographic data

	Female groups		Male groups		F-statistics DEX vs C
	DEX (f)	C (f)	DEX	C (m)	
N	8	24	10	16	
Age mean (SD)	20.62 (2.66)	20.22 (2.41)	20.91 (2.82)	20.99 (2.96)	F(1,56) = 0.116, *P* = .735
Subject education^*a*^ mean (SD)	2.0 (0.41)	1.79 (0.59)	1.90 (0.32)	1.81 (0.54)	F(1,56) = 0.945, *P* = .335
Maternal education^*a*^ mean (SD)	2.38 (0.52)	2.25 (0.68)	2.40 (0.70)	2.31 (0.79)	F(1,56) = 0.344, *P* = .560
Paternal education^*a*^ mean (SD)	2.13 (0.99)	2.30 (0.76)	2.50 (0.71)	2.19 (0.66)	F(1,55) = 0.128, *P* = .722
Wellbeing^*b*^ mean (SD)	6.29 (1.99)	7.51 (0.92)	7.12 (1.81)	7.41 (1.52)	F(1,54) = 3.247, *P* = .077
Nonparametric tests					Total group U-test ^**c**^
Alcohol^*c*^					W = 314, *P* = .745
Drugs^*c*^					W = 369, *P* = .526
Smoking^*c*^					W = 392, *P* = .419

^*a*^Level of education: 1–3 (1: basic, 2: high school, 3: college).

^*b*^General wellbeing: based on a 10-point visual analogue scale.

^*c*^Mann–Whitney U test, exact statistics.

### Procedures

#### Image acquisition.

Anatomical T1 images (176 slices, voxel size: 1 × 1 × 1 mm) and 2 functional magnetic resonance imaging scans during a verbal and visuospatial working memory task (480 volumes, 16 minutes each) were acquired on a 3T Discovery MR750 scanner (GE Healthcare, Little Chalfont, UK), with an 8-channel head coil.

Whole-brain T2*-weighted echoplanar images (TR = 2 seconds, TE = 30 ms, voxel size = 3 × 3 × 3 mm, 41 slices, flip angle 70) were acquired for the functional scans.

#### Working memory tasks.

**Figure 1.**
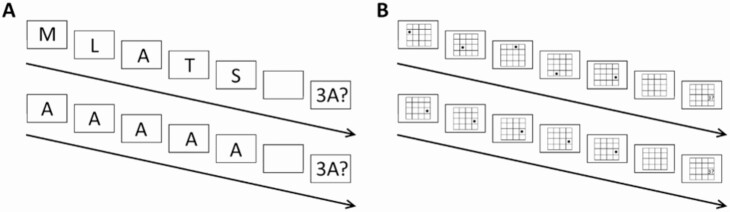
Task setup. Depiction of a single trial of (A) verbal WM task and (B) visuospatial WM task. The top row for A and B represents experimental/working memory trials. Bottom row represents control trials. All displayed trials are probe trials. No-probe trials end after the sixth (empty) screen.

During the functional magnetic resonance imaging acquisitions, participants performed a verbal and visuospatial working memory task with a similar setup, based on a design used in a previous study (18). Participants were shown sequences of 5 items (letters for the verbal task, dot locations on a 4 × 4 grid in the visuospatial task), after which they were asked whether the n^th^ stimuli matched a particular item (eg, “was the third letter an F?”; or “was the third dot in this location?” see Fig. 1). Participants responded by button-press. The experiment consisted of an equal number of pseudorandomized experimental or control trials, with the control trials consisting of the same item being shown 5 times (eg, A-A-A-A-A). Participants were probed on only half of the trials. No more than 3 trials of the same type (control, experimental; probe, no probe) were presented in a row.

Each item (letters, dots) was presented for 500 ms, with a 500 ms “off” period during which a blank screen/empty grid was shown. The fifth item was followed by a jittered interval of 1000 to 2000 ms during which a fixation cross was shown. In trials where participants were asked about a specific item (probe trials), a probe was then shown for up to 3000 ms, or until a response was given. Following the probe, or following the fixation cross period in trials where no item was probed, there was an intertrial interval of 1200 to 3200 ms. Per task, 96 stimuli were shown (48 experimental, 48 control; 24 “probe trials” each). After half of the trials, a 1-minute break was introduced. Therefore, task duration was close to 16 minutes, depending on participants’ response speed.

#### Image pre-processing.

Functional and anatomical images were analyzed with FSL 6.0. Anatomical scans were brain extracted using FSL’s brain extraction tool (BET) (19), segmented into gray matter, WM, and cerebrospinal fluid using FAST (20), and normalized to the Montreal Neurological Institute (MNI) standard brain using deformation fields. Functional images were analyzed using FSL’s FEAT tool for preprocessing and first- and second-level analyses (21, 22). The preprocessing consisted of MCFLIRT motion correction in order to minimize head movements between the images, slice time correction, and coregistration of the functional mean image to the participants’ brain-extracted structural image. In addition, functional images normalization to the MNI template was calculated. Spatial smoothing with a 5 mm full width at half maximum Gaussian kernel was applied to the functional images. Following preprocessing, the functional data were cleaned using ICA-AROMA by removing noise components from the time series with linear regression (23, 24). Those cleaned timeseries were used in the first-level FEAT analyses. Visual inspection of the ICA-AROMA-cleaned images revealed that a few participants had residual motion in a few slices. Therefore, the motion outliers script was run and included as additional confounds in the first-level analyses.

### First-level analyses

The 2 WM tasks were analyzed separately. Two first-level FEAT analyses were run for each experiment (21). In the first, for each trial type (experimental, control), custom wave functions were constructed matching the *encoding* and *decoding* phases of each trial (ie, presentation of the 5 to be remembered items represented the *encoding* phase; presentation of the probe and response represented the *decoding* phase, with the duration of the decoding phase set to 0 to capture the immediate decoding response). In addition, custom wave functions were constructed for the button-press, the rest period in between blocks, and missed responses. All 7 regressors (*encoding*-experimental, *encoding*-control, *decoding*-experimental, *decoding*-control, button press, rest period, missing trials) were convolved with a double gamma hemodynamic response function and its temporal derivative. In the additional first-level FEAT analyses, the encoding and decoding phases were collapsed. As such, 5 regressors (encoding–decoding experimental, encoding–decoding control, button-press, rest period, missing trials) were convolved with the hemodynamic response function and its derivative. Temporal filtering was applied by high-pass filtering with a cut-off of 128 seconds. Parameters estimates were obtained for all regressors by maximum likelihood estimation and a total of 6 (bidirectional) contrasts were calculated pertaining to the effect of working memory load on encoding (encoding experimental vs encoding control) and decoding (decoding experimental vs decoding control), and for encoding and decoding collapsed (experimental vs control), thus creating uncorrected maps with a voxel-wise threshold of *P* < .001.

### Second-level analyses

The parameter estimates obtained from these contrasts were used in a second-level group design using FSL’s randomize tool (10.000 permutations) (22, 25). First, whole group DEX-treated compared with nontreated control analyses were run for all 6 contrasts, and sex and age were included as covariates. In addition, interactions between DEX treatment and sex were assessed, with age as a covariate of no interest. Finally, sex split analyses were run to detect group differences in males and females separately that may have been obscured in the whole-group analyses. The resulting maps were corrected using threshold free cluster enhancement with a *P*-value threshold of <.05. The Harvard–Oxford cortical and subcortical atlases were used to localize significant clusters, and only clusters with a minimum size of 50 voxels are reported.

### Correlations between structure of the left superior frontal gyrus and functional activity and behavior

In our previous study we identified that volume and surface area of the left SFg was increased in DEX-treated non-CAH subjects (n = 19) compared with untreated population controls (n = 43) (16). Here we aimed to test if volume and surface area of this specific region was associated with functional activity during the working memory tasks, and if the structural variation predicted working memory performance in the DEX-treated group. Using FreeSurfer’s qdec tool we drew a region of interest around the area that was significantly different between DEX-treated and controls. A mask was created from the region of interest, which was then back-transformed onto each individual’s image. Next, mean values of cortical surface area and volume were extracted from the masked region in each individual and used in a second-level FEAT analysis to predict activity during encoding and decoding phases of the working memory task, under experimental compared with control conditions, while correcting for sex and age, in the DEX-treated group separately. In addition, values of mean cortical volume and surface area of the left SFg were used in linear regression analyses to predict behavioral outcome as described below.

### Behavioral analyses

Reaction times (RTs) were calculated from correct responses in probe trials and accuracy was measured as the number of correct responses.

### General task effects

First, we analyzed general task effects on RTs and accuracy, by using Student’s t-test to compare performance between the 2 tasks across all participants. In addition, separate t-tests were conducted to test the effect of trial type (experimental vs control) on accuracy and RT on both tasks (verbal and visuospatial) separately.

### Inverse efficiency

Given the possibility of speed–accuracy trade-offs, we also calculated a measure of efficiency, incorporating both reaction speed and accuracy. Efficiency is higher when subjects have higher accuracy at the same RT, or respond with the same accuracy in shorter time. Therefore, inverse efficiency was calculated as RT divided by accuracy.

### Group differences in working memory performance

Linear regression models were used to predict accuracy, RT, and inverse efficiency on the verbal and visuospatial working memory tasks with treatment status (DEX treated or nontreated) while adding sex and age as additional covariates. Next, the interaction between DEX and sex, while controlling for age, was tested on accuracy, RT, and inverse efficiency. These estimates were assessed for experimental and control trials separately, and, in an additional analysis, for experimental and control trials combined. Estimates were considered to be significant at *P* < .05, but we also report estimates with *P* < .1.

## Results

### Behavioral results

#### General task effects.

In general, all participants performed somewhat better as well as faster on the visuospatial task (mean accuracy 44.25 [2.73]; mean RT 912.37 [176.62]) compared to the verbal task (mean accuracy 43.5 [3.21]; mean RT 1160.11 [219.97]) (accuracy: t = 2.274, *P* = .027; RT: t = –14.07, *P* < .001). The experimental (exp) condition resulted in lower accuracy and a longer RT than the control (con) conditions in both the verbal (mean accuracy exp 20.71 [2.21], mean accuracy con 22.79 [1.63], t = –7.292, *P* < .001; mean RT exp 1409.28 [265.41], mean RT con 910.93 [210.82], t = 19.938, *P* < .001) and the visuospatial task (mean accuracy exp 21.28 [1.76], mean accuracy con 22.96 [1.79], t = -5.604, *P* < .001; mean RT exp 1049.93 [208.49], mean RT con 774.80 [167.72], t = 15.306, *P* < .001).

#### Group differences in verbal working memory.

Overall, that is over the experimental and control conditions combined, DEX-treated participants achieved a slightly lower accuracy (C: 44.08 [2.54], DEX-treated: 42.39 [4.22]; B = –1.850, *P* = .041, R^2^ = 0.1319, Cohen’s *d* = 0.485), but not in the 2 conditions assessed separately. In addition, DEX treated had a faster RT on the experimental condition of the verbal task than controls (C: 1460.49 ms [273.21], DEX-treated: 1295.47 ms [212.29]; B = –152.75, *P* = .044, R^2^ = 0.1058, Cohen’s d = 0.675). However, there was no group difference in efficiency (accuracy relative to RT).

#### Group differences in visuospatial working memory.

There were no group differences in any of the estimates of visuospatial working memory, and there were no interactions with sex.

#### Neuroimaging results—verbal and visuospatial working memory.

**Figure 2.**
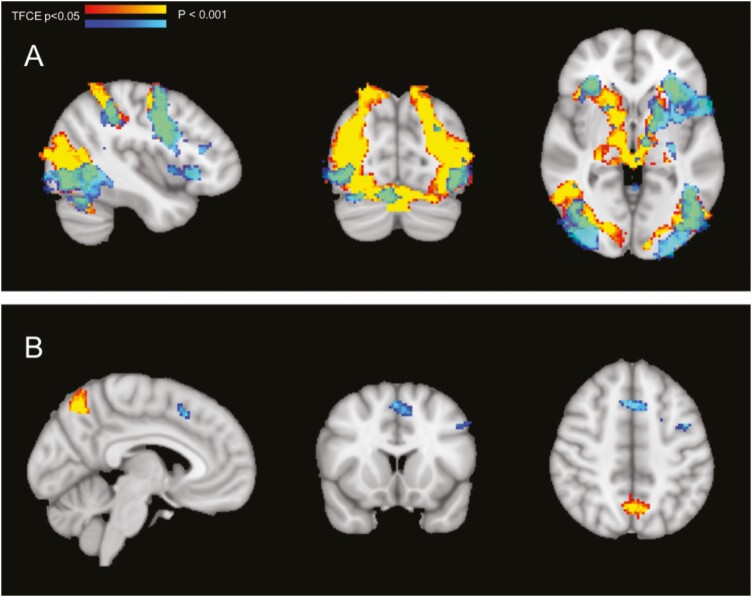
Activation maps of the 2 working memory tasks in participants with DEX treatment. (A) Encoding (exp > con) and (B) decoding (exp > con). Verbal WM task (blue), visuospatial WM task (yellow-red), and the overlap between verbal and visuospatial (green). Activation is limited at z = 3.1.

Both working memory tasks elicited activity in a similar brain network, corresponding to known networks including the occipital and parietal cortexes, precentral gyri, and subcortical regions (Fig. 2), with the visuospatial task eliciting more dorsal occipital and superior parietal areas, corresponding to the dorsal-visual stream. No significant group differences were observed between DEX-treated subjects and controls for any of the main contrasts, for either of the tasks, and there were no interactions with sex.

#### Neuroimaging results—correlations between volume and surface area of the left SFg and functional activity and behavior.

Volume and surface area of the left SFg did not predict behavioral performance on the working memory tasks, see Table 2. Volume and surface area of the left SFg were not associated with brain activity during the encoding or decoding phases of task, when comparing experimental with control conditions.

**Table 2.** Results from the linear regression models testing the association between volume and surface area of the left superior frontal gyrus with accuracy and reaction time on the visuospatial and verbal working memory (WM) tasks in the DEX-treated group (n = 18)

Left SFg Behavior	Left SFg volume			Left SFg surface area		
SPATIAL WM task	B	SE	*P* value	B	SE	*P* value
**Across conditions**						
Accuracy	<0.001	<0.001	.733	0.0012	0.0025	.652
Reaction time	–0.013	0.034	.716	0.057	0.112	.619
**Experimental condition**						
Accuracy	<0.001	<0.001	.927	<0.001	0.0014	.667
Reaction time	–0.009	0.035	.800	0.042	0.114	.719
**Control condition**						
Accuracy	<0.001	<0.001	.695	<0.001	0.0019	.776
Reaction time	-0.016	0.037	.663	0.072	0.121	.559
VERBAL WM task	B	SE	*P* value	B	SE	*P* value
Across conditions						
Accuracy	<0.001	<0.001	.748	0.0021	0.0028	.476
Reaction time	0.012	0.044	.796	0.102	0.142	.484
**Experimental condition**						
Accuracy	<0.001	<0.001	.888	0.0011	0.0014	.467
Reaction time	0.015	0.042	.719	0.096	0.136	.492
**Control condition**						
Accuracy	<0.001	<0.001	.534	0.001	0.0018	.590
Reaction time	0.0077	0.050	.880	0.107	0.161	.517

Results are displayed for the experimental and control conditions separately, and across both conditions. There are no significant associations.

Abbreviations: B, unstandardized regression coefficient; SE, standard error.

## Discussion

The present study assessed brain activity related to working memory performance in healthy participants who had received first-trimester DEX treatment in the context of pregnancies at risk of CAH. Although we observed no differences in brain activity, DEX-treated subjects achieved a slightly lower accuracy across the experimental and control conditions and a faster RT during the experimental condition of the verbal working memory task.

These results are in contrast with our expectation that first-trimester DEX treatment would be associated with altered brain activity during working memory performance in order to achieve similar cognitive performance as controls. The faster RT and slightly reduced accuracy point at a speed/accuracy trade-off in the DEX-treated group. However, the efficiency estimate was not significantly different between groups. Moreover, the lower accuracy was not found in the experimental condition separately, but seemed to be driven by the control condition. Thus, as the faster RT *was* significant for the experimental condition, it seems that DEX-treated subjects performed somewhat better (ie, faster, but not less accurate) than healthy untreated controls on the experimental condition of the verbal task, while achieving similar brain activation.

Previously we reported that first-trimester DEX-treated participants not having CAH performed worse on several cognitive tests as children, with girls being particularly affected, but who seem to catch up during development and do not differ anymore from controls at adult age (14, 15, 26). However, we did find alterations in brain structure even at adult age, which included an enlarged amygdala, larger surface area, and volume of the left SFg, as well as alterations in WM microstructure (16). These alterations in brain structure did not correlate with off-line cognitive performance or behavior. However, due to the involvement of the left SFg in cognitive functions and memory, we had expected that DEX-treated participants would show altered functional activity during working memory performance, especially in this area. Indeed, stimulation of the left SFg, with low-frequency (in the alpha, 8-12 Hz and theta, 3–8 Hz, ranges) direct cortical stimulation via subdural electrodes, has been shown to improve memory performance, specifically by reducing RTs (27). Moreover, the main center of the region that was enlarged in DEX-treated subjects is located in Brodmann area 8, which contains the frontal eye fields and is associated with dealing with uncertainty and the directing of visual attention (28, 29). However, neither volume nor surface area of the left SFg predicted accuracy or RT on the WM tasks in our DEX-treated sample. Thus, neither structure nor function of the brain was associated with behavioral performance. Moreover, structure of these regions did not correlate with functional activity during working memory. Further research is necessary to investigate if the structural alterations are part of a potential compensatory mechanism in DEX-treated subjects and if they are related to improving cognitive performance over time, leading to normalized performance in DEX-treated participants at adult age. Most previous research on prenatal cortisol exposure has assessed the effects on cognition, brain structure, and brain function in childhood and adolescence (8-12). Hence, it would be interesting to investigate if previously reported changes in brain structure and function continue into adulthood and contribute to normalization of cognitive function and behavior later in life.

The mechanisms contributing to the altered developmental pathway remain to be further elucidated, but might include epigenetic alterations. Indeed, earlier we reported changes in DNA methylation of genes involved in the HPA axis, including the glucocorticoid receptor cochaperone *FKBP5* gene, brain derived neurotrophic factor (*BDNF*), the mineralocorticoid receptor (*NR3C2*), and the glucocorticoid receptor (*NR3C1*) (30). Altered methylation of these genes has previously been associated with early life stress (31-33), leading to altered structure of the brain in adolescence (33). Further, in animal studies, prenatal glucocorticoid (DEX) administration has been associated with changes in anxiety and depressive behavior in mice (34), as well as with low basal HPA axis activity in adult rats (35). Rat offspring exposed to DEX have further exhibited memory deficits and anxiety-like behavior, and increased expression of hippocampal glucocorticoid receptors and altered histone acetylation of, among others the *BDNF* gene (36). Interestingly though, prenatal DEX has also shown to increase long-term potentiation in the dentate gyrus in rats (37). These changes correspond to changes in brain structure and gene methylation that we found, but in humans it seems not to lead to a significant change in anxiety, depression, memory abilities, or brain function at adult age. In contrast to contributing to cognitive or behavioral problems for children growing up in adverse circumstances, these changes in DNA methylation and brain structure might turn out to be adaptive in the case of DEX treatment. However, absence of evidence is not evidence of absence, and may be related to the small sample size and relatively simple test.

Despite the unique cohort, some factors need to be taken into consideration when interpreting the results of this study. First and foremost, we only tested brain activity related to this very specific working memory task. In addition, ceiling effects may have prevented us from detecting compensatory mechanisms or difficulties of the DEX-treated brain in coping with the task. Future research is therefore needed to exclude effects of first-trimester DEX treatment on brain function in adults.

Taken together, our findings suggest that, despite problems during childhood and adolescence, prenatal DEX treatment does not lead to significant changes in brain activity underlying working memory performance in adulthood. Adaptive mechanisms cannot be excluded and until now we cannot exclude that the structural alterations that we do observe in limbic structures will not lead to adverse functional or behavioral effects with time. Hence, more research is necessary in additional cohorts, also from other countries, in order to make a well-informed assessment of risks and benefits of prenatal treatment of CAH.

## Data Availability

The datasets generated during and/or analyzed during the current study are not publicly available but are available from the corresponding author on reasonable request.

## Abbreviations

CAH: congenital adrenal hyperplasia
DEX: dexamethasone
GW: gestational week
HPA: hypothalamic–pituitary–adrenal
RT: reaction time
SFg: superior frontal gyrus

## Additional Information

***Disclosure Summary:*** The authors have nothing to disclose.
